# Long-Term Effectiveness and Safety of Biologic and Small Molecule Drugs for Moderate to Severe Atopic Dermatitis: A Systematic Review

**DOI:** 10.3390/life12081159

**Published:** 2022-07-30

**Authors:** Angela Ayen-Rodríguez, José-Juan Pereyra-Rodríguez, Francisco J. Navarro-Triviño, Sara Alcantara-Luna, Javier Domínguez-Cruz, Manuel Galán-Gutiérrez, Samuel Vilar-Palomo, Jose Carlos Armario-Hita, Ricardo Ruiz-Villaverde

**Affiliations:** 1 Dermatology Department, Hospital Universitario San Cecilio, 18016 Granada, Spain; aayenrodriguez@gmail.com (A.A.-R.); fntmed@gmail.com (F.J.N.-T.); ricardo.ruiz.villaverde.sspa@juntadeandalucia.es (R.R.-V.); 2 Dermatology Department, Hospital Universitario Virgen del Rocío, 41013 Sevilla, Spain; javierj.dominguez.sspa@juntadeandalucia.es; 3 Dermatology Department, Hospital Juan Ramón Jiménez, 21005 Huelva, Spain; sara.alcantara.sspa@juntadeandalucia.es; 4 Dermatology Department, Hospital Universitario Reina Sofia, 14004 Cordoba, Spain; manuel.galan.sspa@juntadeandalucia.es; 5 Nursing Department, Faculty of Nursing, Physiotherapy and Podiatry, Sevilla University, 41004 Sevilla, Spain; svilar@us.es; 6 Dermatology Department, Hospital Universitario Puerto Real, 11510 Cádiz, Spain; josecarlos.armario@uca.es

**Keywords:** atopic dermatitis, EASI, IGA, pruritus NRS, systematic literature review, treatment, biologic

## Abstract

Introduction: Atopic dermatitis (AD) is a genetically based chronic inflammatory dermatosis associated with multiple triggers and complex pathophysiological mechanisms. Nowadays, an authentic therapeutic revolution is taking place with the incorporation of biological drugs for the treatment of moderate and severe atopic dermatitis. A new systematic revision (RS) is necessary to support decision-making for specialists treating AD. Methods: A literature search of MEDLINE, EMBASE, and the Cochrane Central Register of Controlled Trials was performed between 1 January 2000 and 30 April 2022. Phase III randomized clinical trials (RCTs) of EMA-approved molecules were included. The main variables analyzed were a 75% improvement in the Eczema Area and Severity Index (EASI 75) and the number of patients who reached 0 in the Investigator Global Assessment (IGA) (fully cleared patients) or IGA 1 (almost cleared patients) at the end of the study period (week 48–60). The risk of bias was analyzed with the Cochrane Risk of Bias Assessment (ROB-2) tool, focused on the primary objectives. Before carrying out the study, the protocol was registered in PROSPERO with the number CRD42022331109. Results: A total of 3299 studies were systematically identified via databases and registers (442 from PubMed/MEDLINE, 2857 from Embase and 719 from CENTRAL). Finally, five publications containing seven RCTs were included in the final sample of detailed data extraction and data analyses. Regarding efficacy, the best results are obtained with Upadacitinib 30 mg (84.7% (77.3–92.1)) at 52 weeks, slightly improving its results when TCS is added (84.9% (80.3–89.5)). These results are replicated in the measurement of vIGA 0/1 for Updacitinib 30 mg + TCS, where 65.5% (55.7–75.2) of patients maintain it at 52 weeks. Of the four drugs, no long-term safety results have been reported for baricitinib. In relation to the safety findings, there were no significant differences in the dropout rates for this reason in the remaining three drugs. Discussion: Today, different therapeutic options for AD patients can be prescribed. Individualizing the treatment allows for better therapeutic consistency, in addition to being cost-efficient to avoid primary therapeutic failures. The results of the present SR may provide us with a useful basis for the preparation of management guidelines for the use of new generation therapies in moderate to severe atopic dermatitis.

## 1. Introduction

Atopic dermatitis (AD) is a chronic inflammatory dermatosis that falls under the concept of immune-mediated inflammatory disease (IMID). With a genetic basis, clinically, it is heterogeneous in its signs and symptoms, although the constant characteristic is the presence of eczema, together with itching and skin xerosis, determining an alteration in barrier function and dysfunction of the immune response towards a Th2 response [1]. It characteristically affects pediatric patients, where most cases appear, affecting around 10–25% of children, although it is also observed in adults, with a prevalence that ranges between 2 and 8% in western countries. Furthermore, almost 25% of adults with AD present with their disease in adulthood [2]. Along with this, it should be noted that in adult patients, a higher percentage of them have a more severe disease [3]. The incidence is higher in women, although in childhood, it predominates in men [4].

Among all the treatments that can be prescribed today, we have topical treatments, where topical corticosteroids represent the “gold standard”, helping with the use of topical calcineurin inhibitors. Currently, there is a true therapeutic revolution taking place with the incorporation of biological drugs and small molecules to treat moderate and severe atopic dermatitis. When the severity of the disease increases, systemic immunosuppressants such as cyclosporine and azathioprine are needed.

The first biological drug incorporated into our therapeutic arsenal is Dupilumab, a humanized monoclonal antibody directed against the IL-4 receptor α subunit, shared with IL-13, thus blocking IL-4/IL-13 and showing encouraging results [5]. Tralokinumab and Lebrikizumab, two monoclonal antibodies directed exclusively against IL-13 have recently been incorporated. They act by competitively blocking the binding of IL-13 to its receptor subunits in B cells and monocytes. Nemolizumab is a humanized monoclonal antibody against IL-31, a key cytokine in pruritus transmission. Tezepelumab is a fully human immunoglobulin G2k monoclonal antibody that binds to thymic stromal lymphopoietin, an epithelial cell-derived cytokine that induces the production of type 2 cytokines, IL-4, IL-5, IL-13, and thyroid factor. Tumor necrosis alpha (TNF-a) is produced by dendritic cells. Janus kinases (JAKs) are enzymes that phosphorylate the intracellular domain of various cytokine receptors. The most recent drugs incorporated into our therapeutic arsenal are precisely JAK antagonists (antiJAK), small molecules administered orally that inhibit cytoplasmic receptors. In AD, several molecules are going to be used. Baricitinib is a selective inhibitor of JAK1/2, while both abrocitinib and upadacitinib are selective inhibitors of Janus kinase 1 (JAK1), which reduces interleukin-4 and interleukin-13 signaling.

Patients with more significant forms of AD will require long-term treatment, and reliable evidence on the comparative benefits and risks of interventions is needed to make appropriate clinical decisions. In this sense, SR are tools that assess the quality of available evidence and the strength of the recommendations for the disease and different treatment alternatives, improving the precision of answering questions not raised by individual studies [6,7]. The incorporation of new therapeutic tools for the treatment of patients with severe AD makes a new SR necessary to support the decision making of specialists who treat AD. In our research group, we have decided to conduct a systematic review that includes drugs in monotherapy and also in combination with TCS (as it is the main form of use in actual clinical practice) that evaluates the results of drugs that have medium-term data (48–60 weeks).

## 2. Material and Method

### 2.1. Search Strategy and Inclusion Criteria

This systematic review was carried out following the criteria included in the 2020 Preferred Reporting Items for Systematic Reviews and Meta-Analyses (PRISMA) guide [8]. Before the study was carried out, the protocol was registered in PROSPERO with the number CRD42022331109. The long-term efficacy and safety of biological and JAK inhibitors in moderate to severe atopic dermatitis, currently approved by the EMA (Dupilumab, Tralokinumab, Baricitinib, Abrocitinib and Upadacitinib), have been analyzed.

A comprehensive literature search was performed using three databases (MEDLINE and EMBASE and the Cochrane Central Register). A search strategy was developed for this purpose and has been included in Supplementary Material S1.

Only studies published in English between 1 January 2000 and 30 April 2022 in human subjects were included.

### 2.2. Study Selection

A first selection was made based exclusively on the title and abstract. This was carried out by two independent researchers (AAR and RRV). Those records that lacked a summary included the full text in order to make the first assessment. Discrepancies were resolved by a third investigator (JPR). The authors then independently evaluated the full text of the studies included in the previous round to determine the final inclusion/exclusion. Dissident articles were resolved by discussion and consensus. In case of persistent disagreement, the same third researcher decided. Neither the journal, authors, nor year was blinded. The inclusion criteria were: published or accepted phase III randomized clinical trials, in English, that included an adult population (over 18 years of age) with moderate to severe atopic dermatitis and whose study period extended to 48–60 weeks. Drugs with RCTs including both adults and adolescents (≥12 years) were also included, indicating the percentage of adolescents. Studies that only included children under 18 years of age were excluded.

### 2.3. Outcomes

The two primary outcomes analyzed were: (a) the number of patients achieving at least a 75% reduction from baseline on the EASI scale (EASI 75) at 48–60 weeks; and (b) the number of patients who reached IGA 0 (fully cleared patients) or IGA 1 (almost cleared patients).

Secondary outcomes included the number of patients who achieved (a) a reduction in at least 90% from baseline on the EASI scale (EASI 90) at 48–60 weeks; (b) an improvement of at least 4 points on the NRS itch scale; (c) the number of patients who experienced at least one AE; (d) the number of patients who experienced at least one SAE.

### 2.4. Assessment of Risk of Bias

**Figure 1:**
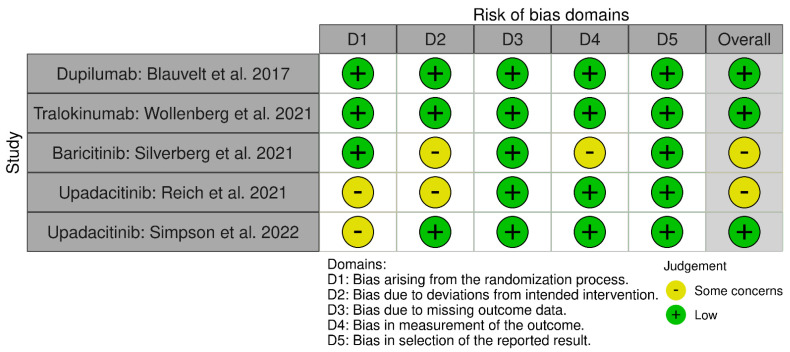
Graphs after the application of version 2 of the Cochrane risk of bias tool for randomized trials (RoB 2): “Graph of risk of bias” [10,11,12,13,14].

**Figure 2:**
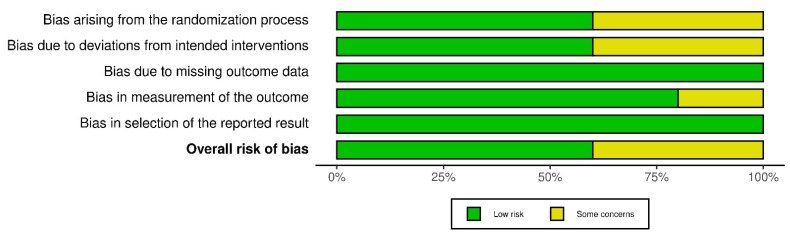
“Summary of risk of bias” (“between the studies”).

Once the selection process of the articles is complete, their methodological quality and the risk of bias of the RCTs are evaluated using the Cochrane Risk of Bias Assessment Tool (ROB-2) [9]. The same three authors carried out this analysis and focused on the main objectives (EASI 75 and IGA 0/1). This tool analyzes bias arising from the randomization process, intervention, data loss, data measurement, and reported results [9]. Each domain will be evaluated by its own algorithm giving a rating of low-risk of bias (low risk), some problems (some concerns), and high risk (high risk). In the same way, the global score of ROB-2 will be graded, being the result of high risk if any of its domains obtains this “high risk” qualification, or of some risk if at least one of them obtains the score of “some concerns”. All are represented in two graphs under the labels “Graph of risk of bias” (Figure 1) and “Summary of risk of bias” (“between the studies”) (Figure 2).

### 2.5. Data Extraction and Quality Assessment

After assessing the methodological quality and risk of bias, qualitative and quantitative data extraction was performed for subsequent analysis. For the descriptive synthesis of the studies, a table of characteristics was prepared that collected the following data. The following data were extracted for the different arms in each study: author(s), year of publication, drug, dose per arm, follow-up (length of study), gender (expressed as percentage of men), race (expressed as percentage of Caucasians), age and weight at inclusion, duration of disease, baseline EASI, baseline body surface area (BSA), number of patients who reached EASI 50, EASI 75, and EASI 90 at the end of follow-up in weeks 48–60, number of patients who achieved IGA 0 or 1 at weeks 48–60, Dermatology Life Quality Index (DLQI) at weeks 48–60, number of patients who achieved a 4-point improvement in the NRS itch scale at weeks 48–60; number of patients with at least one adverse event (AE), number of patients with at least one serious AE (SAE), number of patients with at least one infectious AE, number of patients with at least one upper respiratory tract infection, and number of patients who discontinued treatment due to an AE. Only groups corresponding to approved doses were included.

### 2.6. Strategy for Data Synthesis

According to our study protocol, given the great heterogeneity of the studies, with various drug combinations and re-randomizations, in many cases based on the response obtained after the initial induction period, it was not possible to carry out a network meta-analysis. Therefore, finally, only a literary synthesis of the results obtained in the different included studies was carried out.

## 3. Results

### 3.1. Study Selection

**Figure 3:**
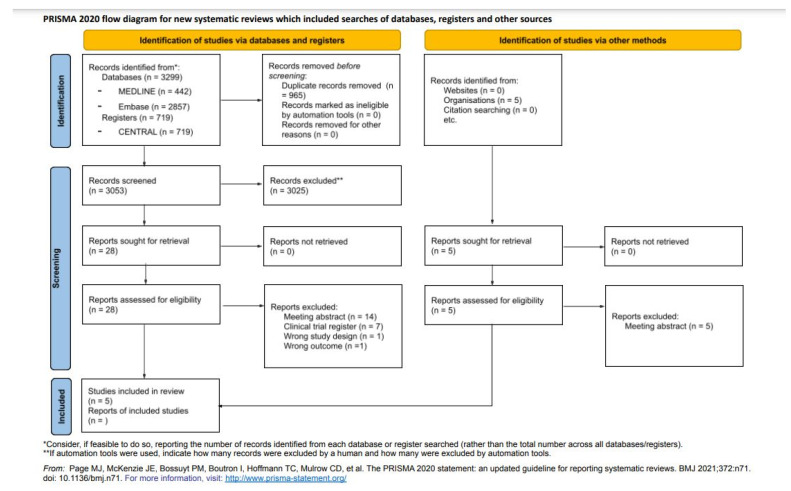
PRISMA 2020 flow diagram for new systematic reviews, which included searches of databases, registers, and others [8].

A total of 3299 studies were systematically identified via databases and registers (442 from PubMed/MEDLINE, 2857 from Embase and 719 from CENTRAL) (Figure 3). After the elimination of duplicate studies, 3053 studies were screened based on titles and abstracts applying eligibility criteria, resulting in 28 studies. In addition, five studies were identified from other sources. These 33 studies were full-text evaluated (exclusion reasons in Figure 3), and 5 publications containing seven RCTs were included in the final sample for detailed data extraction and data analyses.

### 3.2. Baseline Characteristics

Among the selected RCTs, three evaluated the use of Upadacitinib and two evaluated tralokinumab, while dupilumab and baricitinib were evaluated in a single RCT, respectively. No long-term data have been published for the remaining drugs included in the search protocol. Studies testing dupilumab and upadacitinib allowed the use of concomitant TCS with the drug or placebo.

Table 1 [10,11,12,13,14] summarizes the characteristics of the selected studies (date of publication, patients by arm, treatment, dose and route) and the characteristics of the recruited patients. The sample size of the trials analyzed ranged between 124 (BREEZE-AD3) and 901 (AD Up). Mean age ranged between 32.5 and 40.5, and in all the studies, there was a higher proportion of males than females (51.9–64%). Only upadacitinib trials assessed the efficacy and safety of the drug in adolescent patients (including a total of 344 patients). The range of duration of the disease was 18.8–28.0.

Baseline means of EASI and SCORAD scores were 29.1 (range 24.9–30.9) and 62.2 (range 62.2–70.8), respectively, and the mean of proportions of patients with IGA = 4 was 48.2% (range 31.4–55.3).

### 3.3. Efficacy Outcomes

Table 2 [10,11,12,13,14] shows the response achieved in the different variables analyzed in this study. Treatment response was evaluated in all of them by determining the EASI75 and IGA0/1. The rest of the efficacy parameters (EASI50,90,100 and WP-NRS improvement) were not evaluated in all studies. Quality of life measurement using DLQI was only used in two studies.

**Table 1:** Characteristics of the studies and baseline population included in the systematic review.

Publication Data		Study Design				Study Arm Baseline Characteristics										
Study ID	Year	Phase	Agent	Dosing, Schedule, Route	*n*	Males *n* (%)	Age Mean/Median	Adolescent (12–17 Years) *n* (%)	Race (White) *n* (%)	Disease Duration Years Mean/Median	Basal EASI Score Mean/Median	Basal BSA % Mean/Median	Basal SCORAD Score Mean/Median	Weekly WP-NRS Score Mean/Median	vIGA-AD Score = 4 *n* (%)	DLQI Score Mean/Median
LIBERTY AD CHRONOS * [10]	2017	3	Placebo + TCS	QW sc	315	193 (61.3)	34.0 (25.0–45.0)	0	208 (66.0)	26.0 (17.0–38.0)	29.6 (22.2–40.8)	55.0 (40.0–75.0)	64.1 (55.9–76.1)	7.6 (6.3–8.6)	147 (46.6)	14.0 (9.0–20.0)
			Dupilumab + TCS	300 mg Q2W sc	106	62 (58.5)	40.5 (28.0–49.0)	0	74 (69.8)	28.0 (20.0–44.0)	30.9 (22.3–41.6)	58.8 (43.5–78.5)	69.7 (60.4–79.8)	7.7 (6.6–8.5)	53 (50.0)	13.5 (8.0–20.0)
			Dupilumab + TCS	300 mg QW sc	319	191 (59.9)	34.0 (26.0–45.0)	0	208 (65.2)	26.0 (18.0–39.0)	29.0 (21.6–40.7)	52.0 (36.0–71.5)	65.3 (55.2–76.3)	7.4 (6.0–8.6)	147 (46.1)	14.0 (8.0–20.0)
ECZTRA-1 * [11]	2020	3	Placebo	Q2W sc	199	123 (61.8)	37.0 (26.0–49.0)	0	138 (69.3)	28.0 (18.0–41.0)	30.3 (22.0–41.5)	52.5 (31.0–77.0)	70.8 (63.8–81.0)	7.9 (6.9–8.7)	102 (51.3)	16.0 (13.0–22.0)
			Tralokinumab	300 mg Q2W sc	603	351 (58.2)	37.0 (27.0–48.0)	0	426 (70.6)	27.0 (19.0–38.0)	28.2 (21.3–40.0)	50.0 (33.0–70.0)	69.2 (61.5–79.1)	7.9 (6.7–8.9)	305 (50.6)	17.0 (12.0–22.0)
ECZTRA-2 * [11]	2020	3	Placebo	Q2W sc	201	114 (56.7)	30.0 (23.0–46.0)	0	123 (61.2)	25.0 (18.0–36.0	29.6 (20.6–41.4)	50.0 (31.0–74.0)	69.9 (61.9–79.1)	8.1 (7.1–9.0)	101 (50.2)	18.0 (12.5–24.0)
			Tralokinumab	300 mg Q2W sc	593	359 (60.5)	34.0 (25.0–48.0)	0	374 (63.1)	25.5 (17.0–39.0)	28.2 (19.8–40.8)	50.0 (31.0–74.0)	69.5 (60.5–79.1)	8.0 (7.0–9.0)	286 (48.2)	18.0 (13.0–23.0)
BREEZE-AD3 ** [12]	2021	3	Baricitinib	2 mg QD oral	54	28 (51.9)	32.8 (12.7)	0	45 (83.3)	19.2 (11.8)	24.9 (8.7)	NR	62.2 (12.0)	6.1 (2.2)	18 (33.3)	NR
			Baricitinib	4 mg QD oral	70	42 (60.0)	36.7 (15.5)	0	47 (67.1)	23.2 (16.8)	28.1 (10.6)	NR	63.4 (12.3)	6.5 (2.1)	22 (31.4)	NR
AD Up **, *** [13]	2021	3	Placebo	QD oral	304	178 (58.6)	34.3 (12–75)	40 (13.2)	225 (74.0)	24.3 (15.2)	30.3 (13.0)	48.6 (23.1)	NR	7.1 (1.6)	163 (53.6)	16.3 (7.0)
			Upadacitinib + TCS	15 mg QD oral	300	179 (59.7)	32.5 (13–74)	39 (13.0	204 (68.0)	22.9 (13.9)	29.2 (11.8)	46.7 (21.6)	NR	7.1 (1.8)	157 (52.3)	16.4 (7.2)
			Upadacitinib + TCS	30 mg QD oral	297	190 (64.0)	35.5 (12–72)	37 (12.5)	218 (73.4)	23.1 (16.1)	29.7 (11.8)	48.5 (23.1)	NR	7.4 (1.6)	157 (52.9)	17.1 (7.0)
Measure Up 1 **, *** [14]	2022	3	Placebo	QD oral	281 [244]	144 (51.2)	34.4 (12–75)	40 (14.2)	182 (64.8)	21.3 (15.3)	28.8 (12.6)	45.7 (21.6)	66.1 (12.9)	7.3 (1.7)	122 (44.5)	17.0 (6.8)
			Upadacitinib + TCS	15 mg QD oral	281	157 (55.9)	34.1 (12–74)	42 (14.9)	182 (64.8)	20.5 (15.9)	30.6 (12.8)	48.5 (22.2)	68.2 (12.6)	7.2 (1.6)	127 (45.2)	16.2 (7.0)
			Upadacitinib + TCS	30 mg QD oral	285	155 (54.4)	33.6 (12–75)	42 (14.7)	191 (67.0)	20.4 (14.3)	29.0 (11.1)	47.0 (22.0)	67.3 (12.5)	7.3 (1.5)	131 (46.0)	16.4 (7.0)
Measure Up 2 **, *** [14]	2022	3	Placebo	QD oral	278 [241]	154 (55.4)	33.4 (13–71)	36 (12.9)	195 (70.1)	21.1 (13.6)	29.1 (12.1)	47.6 (22.7)	67.9 (12.1)	7.3 (1.6)	153 (55.0)	17.1 (7.2)
			Upadacitinib + TCS	15 mg QD oral	276	155 (56.2)	33.3 (12–74)	33 (12.0)	184 (66.7)	18.8 (13.3)	28.6 (11.7)	45.1 (22.4)	66.6 (12.5)	7.2 (1.6)	150 (54.3)	16.9 (7.0)
			Upadacitinib + TCS	30 mg QD oral	282	162 (57.4)	34.1 (12–75)	35 (12.4)	198 (70.2)	20.8 (14.3)	29.7 (12.2)	47.0 (23.2)	66.7 (13.0)	7.3 (1.6)	156 (55.3)	16.7 (6.9)

Table 2 Data are expressed as *n* (%), median (IQR = interquartile range) * or mean (SD = standard deviation) ** or mean (range) ***. Every 2 weeks, QD = once daily, sc = subcutaneous administration. NR = not reported. EASI = Eczema Area and Severity Index. BSA = body surface area. SCORAD = Scoring Atopic Dermatitis. WP-NRS = Worst Pruritus Numerical Rating Scale. vIGA-AD = validated Investigator Global Assessment for Atopic Dermatitis. DLQI = Dermatology Life Quality Index. Note that the number of patients in the placebo group at the start of the trials in Measure Up 1 (*n* = 281) and Measure Up 2 (*n* = 241) is different from the final sample used to assess efficacy and safety (244 and 241, respectively). However, the baseline patient characteristics listed in this table refer to the baseline sample of each study.

**Table 2:** Efficacy and safety data extracted from the studies included in the systematic review.

Publication Data		Study Design					Efficacy (w52)							Safety (w52)			
Study ID	Year	Phase	Agent	Dosing, Schedule, Route	*n*	*n* 16w	EASI 50	EASI 75	EASI 90	EASI 100	vIGA-AD 0/1	Mean Reduction DLQI	WP-NRS Improvement ≥4	At Least One AE	At Least One Serious AE	At Least One Infectious AE	Withdrawal Due to AE
LIBERTY AD CHRONOS [10]	2017	3	Placebo + TCS	QW sc	E = 264 S = 315		29.9%	21.6%	15.5%	NR	12.5%	−5.6 (0.36)	12.9% (32/249)	266 (84.4)	16 (5.1)	182 (57.8)	24 (7.6)
			Dupilumab + TCS	300 mg Q2W sc	E = 89 S = 110		78.7% *	65.2% *	50.6% *	NR	36.0% *	−10.9 (0.59) *	51.2% (44/86) *	97 (88.2)	4 (3.6)	63 (57.3)	2 (1.8)
			Dupilumab + TCS	300 mg QW sc	E = 270 S = 315		70.0% *	64.1% *	50.7% *	NR	40.0% *	−10.7 (0.36) *	39.0% (97/249) *	261 (82.9)	9 (2.9)	166 (52.7)	9 (2.9)
ECZTRA-1 [11]	2020	3	Placebo		199		NR	NR	NR	NR	NR	NR	NR	NR	NR	NR	NR
			Tralokinumab	300 mg Q2W sc	603	Placebo *n* = 35	NR	33.3%	NR	NR	47.4%	NR	NR	25 (71.4)	0	NR	0
						Q2W *n* = 68		59.6%			51.3%			54 (79.4)	1 (1.5)		1 (1.5)
						Q4W *n* = 76		49.1%			38.9%			53 (69.7)	3 (3.9)		1 (1.3)
ECZTRA-2 [11]	2020	3	Placebo		201		NR	NR	NR	NR	NR	NR	NR	NR	NR	NR	NR
			Tralokinumab	300 mg Q2W sc	593	Placebo *n* = 46	NR	21.4%	NR	NR	25.0%	NR	NR	32 (69.6)	0	NR	0
						Q2W *n* = 91		55.8% *			59.3% *			62 (68.1)	0		2 (2.2)
						Q4W *n* = 89		51.4% *			44.9%			56 (62.9)	3 (3.4)		1 (1.)
BREEZE-AD3 [12]	2021	3	Baricitinib	2 mg QD oral	216	54	NR	81.5%	NR	NR	59.3%	−7.9 (7.9)	NR	NR	NR	NR	NR
			Baricitinib	4 mg QD oral	216	70	NR	55.7%	NR	NR	47.1%	−7.1 (6.7)	NR	NR	NR	NR	NR
AD Up [13]	2021	3	Placebo + TCS Upadacitinib + TCS	15 mg QD oral		144	NR	79.1% (71.7–86.6)	60.8% (51.8–69.8)	27.0% (18.9–35.1)	56.9% (47.8–66.0)	NR	61.3% (52.2–70.3)	338.0 E/100 PY	8.0 E/100 PY	NR	20/443 (4.5)
			Upadacitinib + TCS	15 mg QD oral	300	289	NR	50.8% (45.1–56.5)	37.7% (32.1–43.3)	13.1% (9.2–16.9)	33.5% (28.1–38.9%)	NR	45.3% (39.5–51.0)				
			Placebo + TCS Upadacitinib + TCS	30 mg QD oral		139	NR	84.7% (77.3–92.1)	71.8% (62.2–81.5%)	26.3% (17.3–35.3)	65.5% (55.7–75.2)	NR	70.7% (61.3–80.2)	346.6 E/100 PY	8.1 E/100 PY	NR	20/436 (4.6)
			Upadacitinib + TCS	30 mg QD oral	297	287	NR	69.0% (63.7–74.3)	55.4% (49.7–61.2)	23.6% (18.8–28.5)	45.2% (39.5–50.9)	NR	57.5% (51.8–63.2)				
Measure Up 1 [14]	2022	3	Placebo + TCS Upadacitinib + TCS (w16)	15 mg QD oral		121	NR	NR	NR	NR	NR	NR	NR	262.4 E/100 PY	6.5 E/100 PY	NR	22 (5.5)
			Upadacitinib + TCS	15 mg QD oral	281		NR	82.0% (77.0–86.9)	62.7% (56.5–68.9)	27.9% (22.1–33.7)	59.2% (52.9–65.5)	NR	67.3% (61.1–73.4)				
			Placebo + TCS Upadacitinib + TCS (w16)	30 mg QD oral		123	NR	NR	NR	NR	NR	NR	NR	330.9 E/100 PY	10.0 E/100 PY	NR	39 (9.6)
			Upadacitinib + TCS	30 mg QD oral	285		NR	84.9% (80.3–89.5)	73.3% (67.6–79.0)	35.8% (29.6–41.9)	62.5% (56.3–68.7)	NR	67.7% (61.6–73.7)				
Measure Up 2 [14]	2022	3	Placebo + TCS Upadacitinib + TCS (w16)	15 mg QD oral		120	NR	NR	NR	NR	NR	NR	NR	240.9 E/100 PY	7.1 E/100 PY	NR	21 (5.3)
			Upadacitinib + TCS	15 mg QD oral	276		NR	79.1% (73.9–84.4)	61.3% (55.0–67.6)	27.8% (22.0–33.6)	52.6% (46.2–59.1)	NR	62.4% (56.1–68.7)				
			Placebo + TCS Upadacitinib + TCS (w16)	30 mg QD oral		121	NR	NR	NR	NR	NR	NR	NR	270.9 E/100 PY	6.9 E/100 PY	NR	31 (7.7)
			Upadacitinib + TCS	30 mg QD oral	282		NR	84.3 (79.6–89.0)	70.3% (64.4–76.2)	35.8% (29.6–42.0)	65.1% (58.9–71.2)	NR	72.9% (67.1–78.7)				

* *p* < 0.01 vs. placebo. Efficacy data are expressed as a proportion (95% confidence interval or *n*/N), mean (SD = standard deviation). Safety data are expressed as *n* = number of patients (%) or E/100PY = exposure-adjusted event rate, calculated as the number adverse events divided by the total exposure in 100 patient-years. TCS = topical corticosteroids. QW = once weekly, Q2W = every 2 weeks, QD = once daily, sc = subcutaneous administration, w16 = week 16. NR = not reported. EASI = Eczema Area and Severity Index. BSA = body surface area. SCORAD = Scoring Atopic Dermatitis. WP-NRS = Worst Pruritus Numerical Rating Scale. vIGA-AD = validated Investigator Global Assessment for Atopic Dermatitis. DLQI = Dermatology Life Quality Index. AE = adverse event. Note that some trials use a different *n* for the assessment of efficacy and safety. This is reflected in the table with the following abbreviation: E = *n* for efficacy outcomes, S = *n* for safety outcomes. EZCTRA-1 and EZCTRA-2 reported AEs in the 36-week maintenance treatment period in patients who received tralokinumab in the initial treatment period.

Dupilumab showed significant superior efficacy compared to the placebo for the two coprimary endpoints: IGA 0/1 and two-point or higher improvement in IGA from baseline at week 52 was achieved by 40% and 36% of patients from both dupilumab arms, versus only 13% from placebo arm; and an EASI-75 response at 52 weeks was achieved by 64% and 65% versus 22%.

Maintenance outcomes (IGA 0/1 and EASI 75) at week 52 with tralokinumab were only assessed in patients who achieved IGA 0 or 1 and/or EASI 75 with tralokinumab at week 16 (185/603 and 227/593 patients). These patients were rerandomized to tralokinumab Q2W or Q4W or placebo for 36 weeks. IGA 1-0 was maintained at week 52 in 51% and 47% of patients of tralokinumab groups vs. 47% in the placebo group (EZCTRA 1) and in 59% and 45% vs. 25% (EZCTRA 2, statistically significant differences between tralokinumab Q2W group and placebo group). EASI 75 was maintained by 60% and 49% with continued tralokinumab vs. 33% with placebo (EZCTRA 1) and 56% and 51% vs. 21% (EZCTRA 2).

BREEZE-AD3 shows efficacy results only for patients initially randomized to baricitinib 2 and 4 mg and were classified as responder (IGA 0,1) or partial responder (IGA 2) at week 16 of treatment (patients originating from studies BREEZE-AD1/BREEZE-AD2). Between 54 patients receiving baricitinib 4 mg, the IGA 0/1 proportion remained stable between weeks 16 and 68 of treatment (45.7% and 47.1%), while in patients receiving 2 mg, this proportion increased slightly (51.9% and 59.3%). Results for the EASI75 response were similar in the 2 mg arm, 74.1% at week 16 and 81.5% at week 68; however, in 4 mg arm there was a slight decrease, from 70.0% in week 16 to 55.7% in week 68. Results for other scores such as itch NRI were reported no later than 32 weeks of treatment.

In the three clinical trials evaluating upadacitinib, patients in the placebo group were rerandomized at week 16 to receive upadacitinib 15 or 30 mg, so there are no comparative data on long-term efficacy between placebo and drug. In addition, only AD Up reports efficacy outcomes in placebo to upadacitinib groups. The blinded extension period of AD Up shows a slight decrease in the efficacy parameters assessed in the two upadacitinib arms between weeks 16 and 52. Proportions of patients who achieved IGA 0/1 with UPA 15 mg is 39.3% and 35.0% at week 16 and 52, respectively, and in patients with UPA 30 mg is 58.4% and 45.2%. Similarly, the proportion of patients who achieved EASI75 at week 16 is 64.3% and 76.9% in UPA 15 and 30 mg groups, decreasing at week 52 to 50.8% and 69.0%. However, Measure Up 1 and 2 show greater stability over time in terms of efficacy. Integrated data show an IGA 0/1 for the 15 mg dose of 44.4% and 49.6% at weeks 16 and 52, respectively, while for EASI75, the percentage is 66.0% and 70.2%. Similarly, the 30 mg dose shows a percentage of patients achieving IGA 0/1 of 58.9% and 57.3% at weeks 16 and 52, with an EASI75 of 78.0 and 72.8%.

### 3.4. Safety

Of the four drugs, no long-term safety results have been reported for baricitinib. Overall rates of AEs were similar across dupilumab and placebo groups during the 52-week treatment period. Rates of discontinuations due to adverse events were higher in the placebo group (8% vs. 2–3%); however, 58% of discontinuations were due to atopic dermatitis flares.

The incidence of AEs was comparable between tralokinumab and placebo in the 36-week maintenance treatment period in patients who received tralokinumab in the initial treatment period. The long-term safety of upadacitinib was independent of dose, as both treatment arms (15 and 30 mg) showed a similar rate of AEs. There are no data comparing the placebo at 52 weeks. The rate of treatment discontinuations due to AES was low, between 4.5% and 9.6%, being higher in the upadacitinib 30 mg arm vs. the 15 mg arm.

## 4. Discussion

In recent months, three new molecules have been incorporated into the Spanish Health System for the treatment of AD. This situation has improved the treatment of these patients. A specialist’s decision to start one or the other may be conditioned by the trajectory of the AD (seasonal vs. chronic), associated comorbidities, gestational desire, as well as the short-term and/or long-term efficacy and safety profile of the drug. Therefore, it is essential to compare the four available therapies.

This SR is based on seven RCTs. Three have evaluated the use of upadacitinib, and the other two have evaluated tralokinumab, while dupilumab and baricitinib were evaluated in a single RCT. No long-term data have been published for the remaining drugs included in the search protocol. It should be noted that the studies testing dupilumab and upadacitinib allowed the use of concomitant TCS and only upadacitinib trials assessed the efficacy and safety of the drug in adolescent patients. In routine clinical practice, a combination of TCS and emollients is common and perhaps, therefore, can better reflect the results of daily practice. However, combination therapy studies present an added difficulty of interpretation as it is not easy to decipher which of the results are due to the active drug and which to the TCS. The reduction in the frequency of application and the total consumption (measured in grams) of topical corticosteroids as an adjuvant to new systemic therapies would be interesting. Tralokinumab is currently the only drug that has recorded this aspect, showing a reduction in both variables [15].

Most of the included trials have maintained uniformity regarding the inclusion criteria (with the exception of including some adolescent cases). Thus, we can observe that the baseline characteristics of the different studies show a similar population in severity, age, and time of evolution of AD, etc. The measurement of the efficacy variables also follows homogeneity in included studies.

In all the included studies, the treatment response has been evaluated by determining the EASI75 and IGA0/1. Upadacitinib and dupilumab provided clinically superior efficacy on both parameters. Concomitant use of TCS was allowed in these RCTs. In the SR and NMA carried out, the best efficacy data (EASI75, 52w) are obtained by Upadacitinib 30 mg + TCS, being the main combination that we would use in real clinical practice. These results are confirmed in the percentage of patients who reach and maintain vIGA 0/1 at 52 weeks. Dupilumab and Tralokinumab data are also highly satisfactory.

To compare the molecules studied, it would be convenient to differentiate JAK inhibitors (JAKi) from monoclonal antibodies (mAb). In terms of efficacy, JAKi are usually fast, and the maximum result is achieved in the first 6–8 weeks. However, the response usually decreases gradually in the long term (beyond 16 weeks). A strategy to be evaluated to recover the initial efficacy seems to be the interruption of the drug for a few weeks and reintroducing it. Such a strategy has been observed with other JAKi drugs in hematology (data not shown). Higher doses of JAKi show a greater long-term loss of efficacy when compared to lower doses of the drug. In terms of efficacy at 52 weeks, abrocitinib could not be compared with the rest of the drugs due to the lack of published data.

Regarding the mAb, dupilumab and tralokinumab seem to behave differently from the JAKi. Its response is slower but progressive, reaching the plateau phase of therapeutic response around 26–32 weeks and maintaining the same long-term response (in responders who reach the therapeutic objective, EASI75) beyond 52 weeks of treatment. In terms of efficacy, it might be interesting to calculate the absolute EASI, but it has not been published in any of the studies analyzed.

The safety has been analyzed in terms of adverse effects, severe adverse effects, infections, and withdrawals. Dupilumab and tralokinumab showed the lowest risk of adverse effects; however, most discontinuations were due to atopic dermatitis flares. Ocular adverse effects, mainly conjunctivitis, are characteristic of these drugs; it seems to be inferior in treatment with tralokinumab, although studies in real clinical practice will allow us to better understand its behavior.

Of the JAKi, no long-term safety results have been reported for baricitinib and upadacitinib, and unlike efficacy, it has an independent safety profile despite the dose administered (data not yet published at 52 weeks). Regarding the abandonment of therapy, patients treated with 15 mg of upadacitinib show almost twice as many medication interruptions.

Some risks of bias have been found, but none like the high risk of bias in the “deviation bias” domain, where a study of upadacitinib [16] and another of baricitinib [12] showed some concerns of bias. Other domains with some concerns and risks were “randomization process” in two upadacitinib studies [14,16] and “measurement of the outcome” in a baricitinib study [12]. It must be taken into account that there are differences between the number of RCTs and patients included.

Considering that AD is a chronic disease, long-term studies are interesting, and RCTs included in the present study include data at week 52. The current situation allows for different therapeutic options for patients with AD. Individualizing the treatment allows a better therapeutic consistency, in addition to being cost-efficient to avoid primary therapeutic failures. These results may provide a useful basis for the preparation of treatment guidelines for the use of a new generation of therapies in moderate to severe atopic dermatitis.

## Supplementary Materials

The following supporting information can be downloaded at: https://www.mdpi.com/article/10.3390/life12081159/s1.
