# Supplementary material for: Long-Term Effectiveness and Safety of Biologic and Small Molecule Drugs for Moderate to Severe Atopic Dermatitis: A Systematic Review
Source: Life (Basel). 2022 Jul 30;12(8):1159. doi: 10.3390/life12081159 (PMC9410312; doi:10.3390/life12081159)
Supplement: Supplementary file 1 [file life-12-01159-s001.zip › life-1845164-supplementary.pdf]

PubMed

1. Population:
"Dermatitis, Atopic"[MeSH Terms] OR "atopic dermatitis"[All Fields] OR "atopic eczema"[All Fields] OR "Eczema"[MeSH Terms] OR "Eczema"[All Fields]

2. Intervention:
a. Dupilumab OR dupixent OR REGN-668 OR SAR-231893
OR Nemolizumab OR CIM-331
OR Lebrikizumab OR RG-3637 OR TNX-650 OR MILR-1444A OR PRO-301444
OR Tralokinumab OR adtralza OR CAT-354 OR lp-0162
OR Baricitinib OR Olumiant OR LY-3009104 OR INCB-28050 OR INCB-028050
OR Abrocitinib OR PF-04965842
OR Upadacitinib OR Rinvoq or ABT-494
OR Tezepelumab OR tezspire OR AMG-157
OR Apremilast OR Otezla OR CC-10004 OR AP-506

b. "Biological Therapy"[MeSH Terms] OR "Biological Therapy"[All Fields] OR "Biologic Therapy"[All Fields] OR "Biologic Treatment"[All Fields] OR "Targeted Therapy"[All Fields] OR "Targeted Treatment "[All Fields] OR "Immunomodulatory Therapy"[All Fields] OR "Immunomodulatory treatment "[All Fields]

c. "Antibodies, Monoclonal"[MeSH Terms] OR "Monoclonal Antibodies"[All Fields] OR "Janus Kinase Inhibitors"[MeSH Terms] OR "Janus Kinase Inhibitors"[All Fields] OR "JAK Inhibitors"[All Fields]

a OR b OR c

3. Clinical Trials
"Controlled Clinical Trials as Topic"[MeSH Terms] OR "Randomized Controlled Trials as Topic"[MeSH Terms] OR "random* controlled trial*"[All Fields] OR "random* control trial*"[All Fields] OR "controlled clinical trial*"[All Fields] OR "random* clinical trial*"[All Fields] OR "RCT"[All Fields] OR randomi*[Title/Abstract] OR randomly[Title/Abstract] OR trial[title] OR crossover*[Title/Abstract] OR cross over*[Title/Abstract] OR ((doubl*[Title/Abstract] OR singl*[Title/Abstract]) AND (blind[Title/Abstract] OR blind*[Title/Abstract]))

1 AND 2 AND 3

Embase

1. Population

'atopic dermatitis'/exp OR 'atopic dermatitis' OR 'atopic ezcema' OR 'eczema'/exp OR 'eczema'

2. Intervention:

a. 'dupilumab'/exp OR dupilumab OR dupixent OR regn668 OR 'regn 668' OR sar231893 OR 'sar 231893'
OR 'nemolizumab'/exp OR nemolizumab OR cim331 OR 'cim 331'
OR 'lebrikizumab'/exp OR lebrikizumab OR rg3637 OR 'rg 3637' OR milr1444a OR 'milr-1444a' OR tnx650 OR 'tnx 650' OR pro301444 OR 'pro 301444'
OR 'tralokinumab'/exp OR tralokinumab OR adtralza OR cat354 OR 'cat 354' OR lp0162 OR 'lp 0162'
OR 'baricitinib'/exp OR baricitinib OR olumiant OR ly3009104 OR 'ly 3009104' OR incb28050 OR 'incb 28050' OR incb028050 OR 'incb 028050'
OR 'abrocitinib'/exp OR abrocitinib OR pf4965842 OR 'pf 4965842' OR pf04965842 OR 'pf 04965842'
OR 'upadacitinib'/exp OR upadacitinib OR rinvoq OR abt494 OR 'abt 494'
OR 'tezepelumab'/exp OR tezepelumab OR tezspire OR amg157 OR 'amg 157' OR medi9929 OR 'medi 9929'
OR 'apremilast'/exp OR apremilast OR otezla OR ap506 OR 'ap 506' OR cc10004 OR 'cc 10004'

b. 'biological therapy'/exp OR 'biologic* therap*' OR 'biologic treatment' OR 'targeted therapy' OR 'targeted treatment' OR 'immunomodulatory therapy' OR 'immunomodulatory treatment'

c. 'monoclonal antibody'/exp OR 'monoclonal antibod*' OR 'antibod*, monoclonal' OR 'janus kinase inhibitor'/exp OR 'janus kinase inhibitor*' OR 'jak inhibitor*'

a OR b OR c

3. Clinical Trials

'controlled clinical trial(topic)'/exp OR 'controlled clinical trial*' OR 'controlled trial*' OR 'random* clinical trial' OR 'rct' OR random*:ti,ab,kw OR crossover:ti,ab OR 'phase ii' OR 'phase 2' OR 'phase iii' OR 'phase 3' OR 'phase iv' OR 'phase 4' OR ((doubl*:ti,ab OR singl*:ti,ab) AND (blind*:ti,ab OR mask*:ti,ab))

1 AND 2 AND 3

Cochrane Central Register of Controlled Trials

ID	Search	Hits
#1	MeSH descriptor: [Dermatitis, Atopic] explode all trees	1959
#2	atopic near dermatitis	5234
#3	Atopic dermatitis	5287
#4	Atopic eczema	2470
#5	MeSH descriptor: [Eczema] explode all trees	1159
#6	eczema	4419
#7	#1 OR #2 OR #3 OR #4 OR #5 OR #6	7540
#8	Dupilumab OR dupixent OR REGN-668 OR SAR-231893	670
#9	Nemolizumab OR CIM-331	35
#10	Lebrikizumab OR RG-3637 OR TNX-650 OR MILR-1444A OR PRO-301444	99
#11	Tralokinumab OR adtralza OR CAT-354 OR lp-0162	136
#12	Baricitinib OR Olumiant OR LY-3009104 OR INCB-28050 OR INCB-028050	531
#13	Abrocitinib OR PF-04965842	107
#14	Upadacitinib OR Rinvoq or ABT-494	474
#15	Tezepelumab OR tezspire OR AMG-157	107
#16	Apremilast OR Otezla OR CC-10004 OR AP-506	507
#17	#8 OR #9 OR #10 OR #11 OR #12 OR #13 OR #14 OR #15 OR #16	2583
#18	#7 AND #17 in Trials 706	
